# FAK-Mediated Mechanotransduction in Skeletal Regeneration

**DOI:** 10.1371/journal.pone.0000390

**Published:** 2007-04-25

**Authors:** Philipp Leucht, Jae-Beom Kim, Jennifer A. Currey, John Brunski, Jill A. Helms

**Affiliations:** 1 Department of Surgery, Division of Plastic and Reconstructive Surgery, Stanford University, Stanford, California, United States of America; 2 Department of Trauma, Hand and Reconstructive Surgery, University of Frankfurt/Main, Frankfurt, Germany; 3 Department of Biomedical Engineering, Rensselaer Polytechnic Institute, Troy, New York, United States of America; Dresden University of Technology, Germany

## Abstract

The majority of cells are equipped to detect and decipher physical stimuli, and then react to these stimuli in a cell type-specific manner. Ultimately, these cellular behaviors are synchronized to produce a tissue response, but how this is achieved remains enigmatic. Here, we investigated the genetic basis for mechanotransduction using the bone marrow as a model system. We found that physical stimuli produced a pattern of principal strain that precisely corresponded to the site-specific expression of *sox9* and *runx2*, two transcription factors required for the commitment of stem cells to a skeletogenic lineage, and the arrangement and orientation of newly deposited type I collagen fibrils. To gain insights into the genetic basis for skeletal mechanotransduction we conditionally inactivated focal adhesion kinase (FAK), an intracellular component of the integrin signaling pathway. By doing so we abolished the mechanically induced osteogenic response and thus identified a critical genetic component of the molecular machinery required for mechanotransduction. Our data provide a new framework in which to consider how physical forces and molecular signals are synchronized during the program of skeletal regeneration.

## Introduction

When we understand how to direct the differentiation of stem cells towards specific lineages then theoretically, we will have the ability to regenerate tissues and thus restore the function of damaged or diseased organs. An enormous investment has been made into the identification and characterization of molecular mediators of stem cell self-renewal, proliferation, and differentiation ([1]–[3]; reviewed in [4]) but relatively little attention has been directed towards understanding how physical stimuli influence stem cell fate decisions. Changes in the stiffness of the extracellular matrix can have a profound influence on the fate decisions made by stem cells [5], [6]. For example, stem cells grown on a soft substrate, which replicates the elasticity of brain tissue, adopt a neuronal phenotype whereas cells grown on a stiff substrate, which mimics bone tissue, assume an osteoblast phenotype [7]. These data exemplify the intimate relationship that exists between the behavior of a cell and the extracellular matrix to which it is attached. But how does a cell perceive its extracellular milieu?

Integrin molecules are likely candidates for such mechanosensors because they span the cell membrane and connect at one end to the cytoskeleton and at the other end to the extracellular matrix. In doing so, they fulfill one of the fundamental properties of a mechanosensor, to link the transcriptional machinery of a cell to its outside environment ([8]–[13]; reviewed in [14]). In some biological systems, integrins are converted to a high affinity state in response to a mechanical force, but precisely how physical stimuli are transduced into biological responses via integrins remains poorly understood. Equally puzzling is how integrin-mediated responses are then integrated across multiple cell types and ultimately synchronized into a coordinated tissue-level response in a living organism.

One environment in which these types of questions can be addressed is the bone marrow cavity. Stem cells reside within the bone marrow in a quiescent state, until injury or disease affects the organism. Stem cells become mobilized in response, via an incompletely understood process that involves the activation of multiple signaling pathways (reviewed in [15]). Some of these pathways are activated by physical stimuli, and it was this aspect of stem cell responsiveness that we exploited in our study into the genetic mechanisms underlying mechanotransduction.

## Methods

### Surgical procedure and implant design

All experiments were performed in accordance with Stanford University Animal Care and Use Committee guidelines. Animals were housed in a light- and temperature-controlled environment and given food and water ad libitum. Mice were anaesthetized with an intraperitoneal injection of Ketamine (80 mg/kg) and Xylazine (16 mg/kg) [16]. An incision was made over the right anterior-proximal tibia and the tibial surface was exposed while preserving the periosteal surface. Two screw holes were drilled through both cortices with a high-speed dental engine using a 0.5 mm drill bit. Next, the micromotion device was positioned and fixed with two 0.5 mm titanium Retopins (NTI Kahla GmbH, Germany). Using the center hole of the device as guidance, the mono-cortical implant hole was drilled using a 0.8 mm drill bit. The implant was composed of a surface-characterized polymer (i.e., Poly(L-lactide-co-D,L-lactide; Midwest Plastics, MN) and had a main diameter of 0.8 mm and 0.5 mm-diameter tip that included two circumferential ridges (Medical Micro Machining, Inc., Simi Valley, CA).

A cap was threaded onto the device to assure that the implant was properly positioned and protected from displacement by mouse activity (i.e., chewing). Wounds were closed with size 7-0 Vicryl sutures. Following surgery, mice received subcutaneous injections of Buprenorphine (0.05–0.1 mg/kg) [16] for analgesia and were allowed to ambulate freely. No antibiotics were given, nor were necessary, to any of the animals.

### Micromotion

Micromotion of the implant was generated by a hand-activated system connected to the center column of the bone plate that consisted of a linear variable differential transducer (LVDT; TransTek Inc., Ellington, Connecticut Model #0240-00000), a load cell (Honeywell Sensotec, Columbus, Ohio Model #11), a DaqBook system (Iotech Inc., Cleveland, Ohio), and a core for the LVDT. One end connected to the load cell, and the other consisted of a 1 mm tip that passed through the cap of the bone plate to produce axial micromotion with a 1.0 Hz frequency, a 60 sec duration, and a 24 h interval, for a period of three, seven or fourteen days depending upon the experiment.

### Tissue processing, histology and immunohistochemistry

Following euthanasia, the treated limbs were dissected, removed of their epidermis and fixed in 4% paraformaldehyde overnight. Decalcification was achieved by introducing the samples into 19% EDTA-2Na solution for two weeks at 4°C. After demineralization, the implant device was gently pulled out of the bone. Specimens were dehydrated through an ascending ethanol series prior to paraffin embedding. Eight micron-thick longitudinal sections were cut of all samples and collected on Superfrost-plus slides for histology using a modification of Movat's Pentachrome staining [17] and aniline blue staining. Immunohistochemistry for PCNA (Zymed) [18] was performed on adjacent slides, using DAB (Zymed) as substrate. In situ hybridization was performed using digoxigenin-labeled probes synthesized complementary to mouse cDNAs for *sox9, runx2*, and *col1* [19]. Tissues were stained with the acidic dye, picrosirius red, to discriminate tightly packed and aligned collagen molecules. Under polarized light, well aligned fibrillar collagen molecules present polarization colors of longer wavelengths as compared to less organized collagen fibrils that show colors of shorter wavelengths.

### FAK inactivation

We generated the conditional knock-out of focal adhesion kinase (FAK) by crossing Cre mice in which the transgene was driven by a 2.3Kb osteoblast-specific *Col1a1* promoter with mice carrying a floxed *fak* allele [20]–[22]. The Col1Cre^+/+^;FAK^fl/fl^ (FAK mutant) genotype was confirmed by PCR. The conditional knock-out was defined by the presence of the *cre* and the absence of the second FAK kinase domain.

### Finite element modeling

To further study the strain environment in tissues surrounding the implants, we developed 2-dimensional and axisymmetric finite element (FE) models using ABAQUS/CAE version 6.6. The assumed geometry for the modeling was as follows: (1) pin implants were modeled with a tip diameter of 0.5 mm; (2) the size of the gap between the implant and the surrounding bone was 0.15 mm; (3) the distance from the base of the implant to the bottom of the other cortex was an average of 0.50 mm; (4) the width of the bone on either side of the interface was 0.725 mm; and (5) the implant was axially displaced 0.15 mm. Simulations were run using different elastic properties for the interfacial tissue while the properties for the bone (E = 11 GPa, ν = 0.426) and PLA implant (E = 432 MPa, ν = 0.35) remained unchanged. The mechanical properties used in the modeling were as follows. To simulate our *in vitro* tests with a rubbery interface marked with tantalum powder, we used a Young's modulus E = 1.2 MPa and Poisson's ratio ν = 0.49. For an interface containing fibrin and cells, E = 19 kPa [23] and Poisson's ratio ν = 0.25. For interfacial cartilage, the assumed elastic modulus was E = 10 MPa, with Poisson's ratio v = 0.167 [24], [25] or v = 0.463 [26].

### Strain measurements using digital image correlation

In estimating the strain fields in the *in vivo* environment of the skeletal injury site surrounding the pin, we made a first approximation using an *in vitro* test system. The micromotion system was attached to a small wooden dowel with the test pin residing in a 0.8 mm diameter hole filled with Reprorubber (Small Parts, Inc., Miami Lakes, FL) and fine tantalum powder. The purpose of the tantalum powder was to provide radio-opaque fiducial markers that could be followed using µ-CT images for the purpose of strain analysis.

The wood dowel with the micromotion device was placed in the micro-CT with the long axis of the tibia running vertically. µ-CT scans were done before and after implant displacement in the rubber/tantalum powder interface. The stage of the µ-CT scanner allowed 360° rotation of the wooden dowel about its long axis in small angular steps of ∼0.5°. Images had a resolution of approximately 1024×1024 pixels; with a pixel size of 5.959 µm. Images were further processed in Analyze software. The center plane of the implant was defined by stepping through the slices, which were 6 µm apart, to find where the pin exhibited its widest diameter. Pre- and post-displacement images were then analyzed via DISMAP [27] to determine the strain fields around the implant.

### Histomorphometric measurements

Tibiae were collected on post-surgical d7 to determine the volume of new bone in the marrow cavity. After paraffin embedding and sectioning, tissues were stained with Aniline blue, and representative sections were analyzed as described below. The implant region in each condition (i.e., wild type stationary, stimulated; FAK mutant stationary, stimulated) was represented across approximately 40 tissue sections, each of which was 8 µm thick. Out of those 40 sections, 6–8 tissue sections were used for histomorphometric measurements. Each section was photographed using a Leica digital imaging system (5× objective). The digital images were imported into Adobe Photoshop CS2. The region of interest typically encompassed 10^6^ pixels. The number of Aniline blue stained pixels was determined using the magic wand tool (tolerance setting; 60, histogram pixel setting; cache level 1) by a single blinded investigator, and confirmed by a second independent investigator. These data were then used to calculate the total volume of new bone in each bone marrow cavity.

### Statistical analysis

Data are given as mean±s.e.m. Group mean values were compared by the student's t-test.

## Results

**Figure 1:**
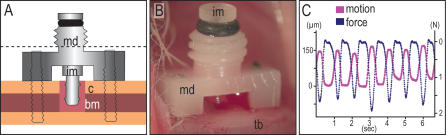
*In vivo* implant device permits defined stimulation of the bone marrow tissue. (A) A motion device, consisting of an intra-osseous, pin-shaped implant (im), held in place by a subcutaneous fixation plate is secured to the mouse tibia by two screws (dotted line is approximate skin level). An O-ring placed between the head of the implant and the center column of the fixation plate acts as a spring to return the implant to its starting position after axial displacement. (B) *In vivo* setting of micromotion device on murine tibia. (C) A linear variable differential transducer (LVDT) and load cell connected to the implant head and fixation plate allows the application and recording of displacement (∼150 µm) and the force (∼1N) required to produce motion.

We accessed the adult bone marrow cavity by creating a small pinhole in the tibial cortex. A permanent device was then attached to the tibia, which consisted of an implant that projected a short distance into the bone marrow cavity (Fig. 1A,B). The implant was moved with an external adaptor that recorded the magnitude of the displacement and the force required to produce this displacement (Fig. 1C). We developed a regimen for mechanical stimulation that consisted of a 60-sec period of axial implant displacement performed at a frequency of 1 Hz. This protocol was repeated once per day.

**Figure 2:**
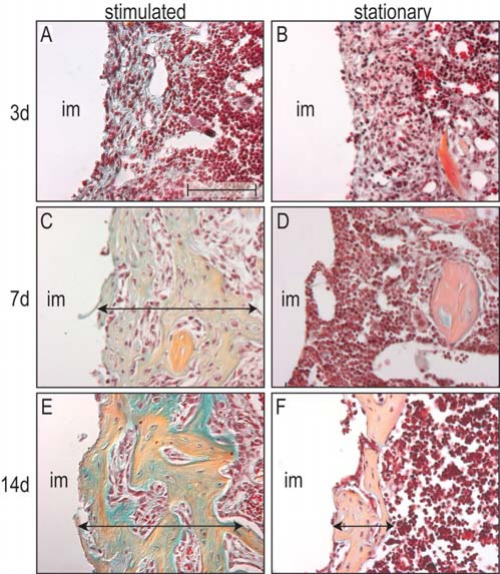
Mechanical stimulation expands the pool of osteoprogenitor cells and accelerates their differentiation into osteoblasts. (A) On post-surgical d3, cells in the stimulated peri-implant space are densely packed within a proteoglycan-rich extracellular matrix (blue). (B) In the stationary environment, cells are loosely organized with no evidence of a mineralized extracellular matrix. (C) By d7, a thick (250 µm), fully mineralized bony sheath encapsulates the stimulated implant. (D) The tissue surrounding the stationary implant is absent of any bone matrix. (E) By d14, the bony encasement is more organized and still retains its original thickness. (F) The stationary tissue exhibits first sign of mineralization (90 µm thick) after 14 days. Scale bar: 100 µm.

Within 72 h of initiating mechanical stimulation we found that bone marrow cells adjacent to the displaced implant exhibited a very subtle yet reproducible increase in proteoglycan-rich extracellular matrix (n = 5; Fig. 2A,B). These differences were amplified with time, so that after 7d, bone marrow cells subjected to mechanical stimulation had differentiated into osteoblasts (n = 8; Fig. 2C). In comparison, unperturbed bone marrow cells retained their fibroblastic appearance (n = 8; Fig. 2D). After 14d, mechanically-stimulated bone marrow cells had differentiated into osteocytes that were encased in a mature bony matrix interlaced with blood vessels (n = 5; Fig. 2E). Cells in the unstimulated marrow environment eventually differentiated into osteoblasts as well, but they did so in far fewer numbers, and only after considerable delay (n = 5; Fig. 2F).

**Figure 3:**
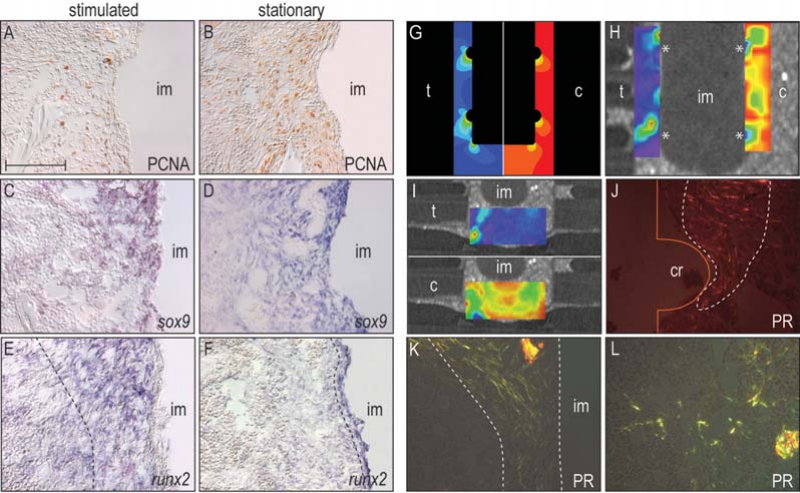
Molecular and cellular response mirrors strain pattern. (A,B) PCNA staining reveals no differences in cell proliferation between unloaded and loaded samples. (C) In stimulated and (D) stationary implants *sox9* is diffusely expressed throughout the surrounding bone marrow cavity. (E) *Runx2* is broadly and strongly expressed in the peri-implant region in unstimulated samples, (F) whereas physical stimulation induces restriction of the *runx2* transcripts to the cells adjacent to the implant. (G) Finite element modeling shows strain concentrations (tensile strain (t), compressive strain (c)) at the circumferential ridges and at the bottom of the implant (for illustration purposes, tensile strains were plotted on the right and compressive strains on the left). (H,I) µCT was used to record displacement of Tantalum particles, and principal strains were calculated by digital image correlation. Implant displacement generated a range of strain fields concentrated around circumferential ridges (cr)(*). (J,K) Picrosirius red staining in conjunction with polarized light microscopy reveals that in loaded samples, the peri-implant collagen fibrils (yellow-red) are abundant, tightly packed, and aligned parallel to the displacement trajectory, (L) whereas in unloaded samples, the collagen fibrils are unorganized. Scale bar: 100 µm.

We initially hypothesized that the basis for this mechanically-induced osteogenesis was enhanced cell proliferation in the stimulated bone marrow, but an examination of proliferating cell nuclear antigen (PCNA) immunostaining did not support this interpretation. The number of PCNA-positive cells in the stimulated and stationary bone marrow cavities was nearly equivalent at the 72 h time point (Fig. 3A,B). We also performed *in situ* hybridization for two transcription factors whose expression predicts the skeletogenic fate of cells. *Sox9* is expressed by all osteochondroprogenitor cells [28], [29] whereas *runx2* is up regulated in cells that have initiated differentiation into an osteoblast lineage ([30]; reviewed in [29]). We found that *sox9* was widely expressed in both stimulated and stationary bone marrow cavities (Fig. 3C,D), indicating the presence of osteochondroprogenitor cells in both sites. A subset of *sox9*-positive cells also expressed *runx2*, and it was this expression domain that was altered by mechanical stimulation. Whereas *runx2* was restricted to a narrow band of cells adjacent to stationary implants (Fig. 3F), cells throughout the stimulated bone marrow cavity expressed *runx2* (Fig. 3E).

These results provided us with an important insight into how mechanical stimulation altered the fate of cells: the very broad expression domain of *runx2* indicated that even cells located at a considerable distance from the implant could sense the physical stimulus. When an implant is displaced within a pliable material such as the bone marrow cavity, then this displacement results in deformation of the extracellular matrix. A convenient measure of deformation is strain, and analogies have been made between strain fields and morphogen fields since both can act over considerable distances to influence cell fates [31]–[33]. We first carried out a finite element analysis to predict the strain pattern and found that the highest principal strains occurred around the circumferential ridges and at the base of the implant (Fig. 3G). Changing the material properties of the bone marrow cavity from a low to a high modulus of elasticity had little effect on the strain pattern (data not shown). Next, we created an *in vitro* model, in which we replicated the material properties of the rigid cortical bone and the pliable marrow cavity. We used µCT images to determine the position of fiduciary landmarks in the simulated peri-implant tissue before and after displacement of the implant, and then used these data points to calculate both the magnitude and the pattern of the strain fields (Fig. 3H,I). Once again, displacement generated a pattern where the highest strains were located around the circumferential ridges and the base of the implant and lower strains were found elsewhere (Fig. 3H,I).

Both the modeling results and *in vitro* stimulation showed that strains were concentrated in discrete locations around the implant. Did this strain pattern have a biological correlate? One mechanism by which strain fields can influence cell behavior is by altering their extracellular matrix. We re-examined the bone marrow cavity using picrosirius red staining and polarized light and found that collagen fibrils in the extracellular matrix were fully aligned and oriented parallel to the direction of displacement (Fig. 3J,K). In the absence of displacement, collagen fibrils were randomly organized (Fig. 3L). The strain pattern and the arrangement of the collagenous matrix showed a one-to-one correspondence, which was clearly evident in the area around the circumferential ridges (compare Fig. 3G,I with Fig. 3J,K).

Thus far, our data demonstrate that even a brief physical stimulus is sufficient to induce the rearrangement of the extracellular matrix of the bone marrow cavity. This pattern of collagen fibril organization mirrors the pattern of strain created by implant displacement. In response to implant displacement, bone marrow cells up-regulated an osteogenic gene, *runx2*. Within a few days the bone marrow cells have differentiated into osteoblasts. But how do bone marrow cells initially detect the deformation of the extracellular matrix, which triggers this mechanically-induced osteogenic response?

**Figure 4:**
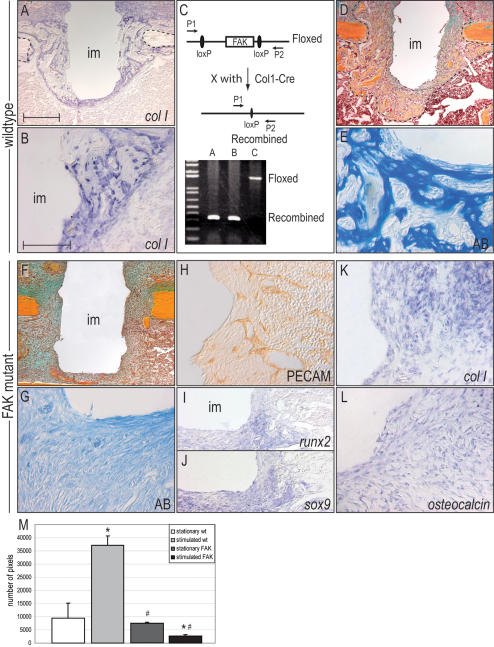
FAK inactivation specifically blocks mechanically induced osteogenesis *in vivo*. (A) *Col I* expression marks peri-implant cells, (B) including those juxtaposed to the implant (im). (C) The schematic indicates the genomic structure of floxed FAK mice; crossing these mice with Cre mice carrying a 2.3Kb osteoblast-specific *Col1a1* promoter resulted in Col1Cre^+/+^;FAK^fl/fl^ (FAK mutant) mice. PCR was used to identify deletion of the *fak* allele in the animal. (D) In wildtype animals, seven days of stimulation result in abundant bone formation. (E) High magnification (Aniline blue) shows newly deposited bone matrix (blue) interlaced with blood vessels. (F) In FAK mutant mice, mechanical stimulation failed to induce osteogenesis. Note that FAK mutants were able to regenerate bone in unstimulated regions, as seen on the right periosteal surface. (G) Aniline blue staining shows complete absence of mineralized tissue in the peri-implant site. (H) Vascular ingrowth is not impeded by the deletion of FAK. (I,J,K,L) FAK mutant cells express *sox9, runx2, col I* and *osteocalcin* indicating that loss of FAK does not hamper the recruitment of osteochondroprogenitor cells to the peri-implant site. (M) Quantitative histomorphometric assessment of newly deposited bone matrix in unstimulated wild type (wt) bone marrow cavities (white), in stimulated wt bone marrow cavities (light gray), stationary FAK mutant bone marrow (gray), and in stimulated FAK mutant bone marrow cavities (black). * (P<0.1), # (p<0.001) indicates significant difference. Scale bar in A,D and F: 300 µm; in B,E and G–L: 100 µm.

There are likely to be a variety of mechanisms by which a cell senses a change in its extracellular matrix and then transduces the physical stimulus into a biological signal. We devised a genetic approach to specifically test if integrin-mediated signaling was essential for mechanotransduction in the bone marrow cavity. Multiple integrins are implicated in mechanotransduction [14]; therefore rather than deleting the structural protein itself we inactivated focal adhesion kinase (FAK), a tyrosine kinase that is involved in signal transduction from integrin-enriched focal adhesion sites. We took advantage of the fact that *collagen type I* is expressed in bone marrow cells around the implant (Fig. 4A,B). Crossing Col1Cre transgenic mice [21] with mice carrying a floxed *fak* allele [22] resulted in the conditional inactivation of FAK in bone marrow cells surrounding the implant (Fig. 4C). Previous *in vitro* and *in vivo* studies have shown that FAK mutant mice are able to secret a mineralized matrix and regenerate skeletal defects [20]. Our histomorphometric measurements showed that FAK mutants formed bone around a stationary implant comparable to their wild type counterparts (Fig. 4M).

We subjected Col1Cre^+/+^;FAK^fl/fl^ mice and their wild type counterparts to the implant displacement protocol and as expected, wild type mice showed an exuberant osteogenic response to mechanical stimulation (n = 7; Fig. 4D,E). In contrast, mechanical stimulation elicited no osteogenic response from FAK mutant bone marrow cells (n = 8; Fig. 4F,G,M). FAK mutant mice showed 93% less bone matrix then their wild type counterparts. The only evident matrix deposition occurred on the periosteal surface in distance from the mechanical stimulus. In the bone marrow cavity, there was no evidence of a mineralized matrix (Fig. 4G), despite the normal vascularization (Fig. 4H) and expression of osteochondroprogenitor cell marker, such *sox9* and *runx2* (Fig. 4I,J). Even terminal osteoblast differentiation marker, like *collagen type I* and *osteocalcin*, were expressed in FAK mutants. Thus, FAK deletion did not prevent osteoblast differentiation. Rather, FAK inactivation specifically blocked the ability of bone marrow cells to sense a mechanical stimulus and reproducibly and rapidly repressed osteoblasts from depositing a mineralized matrix.

## Discussion

All non-circulating cells are equipped to detect and decipher physical stimuli, and then react to these stimuli in a cell type-specific manner. Ultimately, these cellular behaviors are synchronized to produce a tissue response, but how this is achieved remains enigmatic.

Here, we investigated the genetic basis for mechanotransduction using the bone marrow cavity with its high number of stem cells as a model system. We found that physical forces triggered the site-specific expression of *sox9* and *runx2*, two transcription factors required for the commitment of stem cells to a skeletogenic lineage. This physical force produced a pattern of effective strain that precisely corresponded to the arrangement and orientation of newly deposited type I collagen fibrils in the bone marrow cavity. To gain insights into the genetic basis for skeletal mechanotransduction, we conditionally inactivated focal adhesion kinase (FAK), an intracellular component of the integrin signaling pathway. As a consequence of FAK deletion, the cellular response to physical stimuli was abolished: bone marrow cells no longer up-regulated skeletogenic genes, collagen fibrils remained disorganized, and the mechanically-induced osteogenic response was lost. Collectively, these data provide *in vivo* evidence for the basis of mechanotransduction in the bone marrow cavity, and that skeletal progenitor cells detect physical stimuli.

The mechanical environment plays an equally critical role during skeletal tissue repair [34], where micromotion is sometimes viewed as an osteoinductive stimulus [35] but excessive, uncontrolled motion leads to delayed fracture healing, skeletal non-unions, bone graft failures, and implant loosening [35], [36]. This extraordinary mechanosensitivity of the skeleton may be attributable in part to the variety of skeletal cells whose behavior is influenced by physical stimuli. For example, osteoclastogenesis itself may be inhibited by physical stimuli [37] but matrix remodeling by mature osteoclasts is enhanced [38]. Osteocytes are highly responsive to fluid flow changes within the canalicular network [39], [40] while osteoblasts react to deformations in their collagen-rich extracellular matrix [41]. Osteochondroprogenitor cells in the bone marrow stroma are mechanosensitive [42]. Therefore, understanding the skeleton as a mechanosensitive organ is predicated upon appreciating the heterogeneity of mechanosensitive cells mediating bone formation and remodeling, and being aware of the inherent variability in cellular response to the same physical stimulus [33], [43]. The physical stimulus itself is also a source of unpredictability since force transmission can be quantified to some degree, but determining how a cell experiences a mechanical stimulus depends upon variables such as substrate stiffness and the type (e.g., compressive, tensile) and magnitude of the resulting strain (reviewed in [44]).

We developed a model system to explore the process of mechanotransduction, whereby a physical stimulus is converted into a biological response. We chose a model of skeletal tissue regeneration because of the well documented mechanosensitive properties of the skeleton [45], and in so doing, gained critical insights into how skeletal progenitor cells sense a mechanical force, interpret these forces, and then respond by altering their behavior. Our mathematical modeling predicts that implant displacement creates strain fields within the surrounding tissues (Fig. 3G–I) and that cells migrating into this wound environment align themselves along the strained extracellular matrix and that the collagen fibrils secreted by these cells also become oriented parallel to the strain trajectories (Fig. 3J,K). A fraction of these cells are osteoprogenitors, based on their co-expression of *sox9* and *runx2* (Fig. 3C,E) and these cells appear to exploit the collagen rich matrix to sense their mechanical environment. Osteoprogenitors are able to do this by attaching to the extracellular matrix via their cell surface integrins, and then are dependent on the molecular machinery of the focal adhesion to transduce this mechanical stimulus into a biological signal (Fig. 4F,G).

A number of questions remain. For example, finite element models [35], [46] and *in vitro* studies [47] predict the existence of osteogenic and chondrogenic strain fields. These strain characteristics could be recapitulated *in vivo* using this device, which would then enable direct testing of the osteogenic and chondrogenic strain hypotheses. Another variable that remains to be explored is the extent to which a cell's response can be altered by changing the surface characteristics of an implant. Do modifications such as nano-texturing and growth factor coating stimulate osteogenesis *in vivo*? By examining the spatiotemporal patterns of osteogenic gene expression one may be able to directly compare the osteoinductive or osteoconductive properties of such surface modifications (reviewed in [48]). In the broader context of skeletal regenerative medicine, a clear connection exists between the mechanical environment and the differentiation of skeletal progenitor cells into chondrocytes or osteoblasts. For example, the early, controlled loading of fractures accelerates bone healing but for unknown reasons. Are there specific physical stimuli that enhance skeletal progenitor cell proliferation, thereby creating a larger pool of cells that contribute to the regenerate? Or are these physical forces beneficial in the formation of a vascular network, which in turn supports osteoblast differentiation? Finally, there are subtleties to tissue mechanics that are difficult to capture using *in vitro* systems. For example, the length and time scales of physical stimuli that have greatest relevance to cell sensing and cell behavior are difficult to estimate, and while one can now measure forces exerted by individual cells, it is nearly impossible to extrapolate this to groups of cells, tissues, and organs. By determining how skeletal progenitor cells sense and respond to mechanical stimuli, we will undoubtedly find clues as to how to optimize physical stimuli to accelerate skeletal tissue regeneration.
